# Ouabain alleviates *Mycobacterium abscessus*-triggered inflammatory responses through dual regulation of NLRP3 inflammasome activity and M1 macrophage polarization

**DOI:** 10.3389/fimmu.2025.1633882

**Published:** 2025-08-15

**Authors:** Nan Li, Songqiang Huang, Xing Shi, Kuo Lu, Xiu Yu, Chen Qiu, Rongchang Chen

**Affiliations:** ^1^ The Key Laboratory of Shenzhen Respiratory Diseases, Institute of Shenzhen Respiratory Diseases, The First Affiliated Hospital (Shenzhen People’s Hospital), School of Medicine, Southern University of Science and Technology, Shenzhen, China; ^2^ College of Food Science and Pharmaceutical Engineering, Zaozhuang University, Zaozhuang, Shandong, China; ^3^ Department of Pharmacology, Joint Laboratory of Guangdong–Hong Kong Universities for Vascular Homeostasis and Diseases, School of Medicine, Southern University of Science and Technology, Shenzhen, Guangdong, China; ^4^ Henan International Joint Laboratory of Children’s Infectious Diseases, Children’s Hospital Affiliated to Zhengzhou University, Henan Children’s Hospital, Zhengzhou Children’s Hospital, Zhengzhou, China

**Keywords:** *Mycobacterium abscessus*, ouabain, pulmonary inflammation, M1 macrophage polarization, NLRP3 inflammasome activation

## Abstract

**Introduction:**

*Mycobacterium abscessus* (*M. abscessus*) is a highly drug-resistant pathogen responsible for chronic pulmonary inflammation in humans. The cardiac glycoside ouabain exhibits broad anti-inflammatory effects in various disease models, but its therapeutic potential against *M. abscessus*-induced pneumonia remains unexplored.

**Methods:**

We investigated the role of ouabain in *M. abscessus*-induced inflammation using *in vivo* and *in vitro* models. Inflammatory responses were assessed through cytokine expression analysis (TNF-α, IL-6, IL-1β), histopathological examination (H&E staining), transcriptomic profiling, IHC, TEM and qPCR. The effects of ouabain on NLRP3 inflammasome activation and macrophage polarization were also evaluated.

**Results:**

Ouabain significantly reduced *M. abscessus*-induced inflammation by suppressing proinflammatory cytokines (TNF-α, IL-6, IL-1β) and attenuating lung tissue damage. Transcriptomic and qPCR analyses confirmed that ouabain downregulated NLRP3 inflammasome activity and IL-1β secretion *in vivo*. *In vitro* studies further demonstrated that ouabain inhibited NLRP3 inflammasome activation and M1 macrophage polarization.

**Discussion:**

These findings indicate that ouabain mitigates *M. abscessus*-induced pulmonary inflammation through dual mechanisms: suppression of NLRP3 inflammasome activation and modulation of M1 macrophage polarization. This study highlights ouabain’s potential as a therapeutic candidate for *M. abscessus* infections.

## Introduction

Nontuberculous mycobacteria (NTM) have emerged as significant human pathogens, causing chronic and debilitating pulmonary diseases that have become a global health concern in several countries (1). Among the approximately 200 identified NTM species, which are classified as either rapidly growing mycobacteria (RGM) or slowly growing mycobacteria (SGM) (2). Epidemiological studies indicate that RGMs account for approximately 5% of pulmonary infections, while *M. abscessus* complex is responsible for 65-80% of these RGM cases in regions such as Japan and North America (3). As the second most prevalent causative agent of NTM pulmonary disease (NTM-PD), *M. abscessus* can lead to both pulmonary and extrapulmonary manifestations (4). Clinically, *M. abscessus* infections predominantly affect immunocompromised individuals and patients with underlying pulmonary conditions such as cystic fibrosis (CF), chronic obstructive pulmonary disease (COPD), and bronchiectasis (5, 6). Other possible manifestations are main keratitis, endophthalmitis, cerebral abscesses, and meningitis (7, 8). *M. abscessus*-induced chronic infection is associated with a significant decline in lung function and a profound impact on a patient’s quality of life (9). However, the treatment for *M. abscessus* infection remains ineffective, with success rates as low as 30% due to intrinsic, adaptive, and acquired resistance (10). These therapeutic challenges underscore the urgent need for a better understanding of *M. abscessus* pathogenesis and the development of novel treatment strategies.

The host immune response to *M. abscessus* centers on macrophages, which serve as both primary defense cells (11) and intracellular niches for bacterial survival (12). Upon microenvironment stimulation, macrophages can be polarized into pro-inflammatory M1 phenotype and anti-inflammatory M2 phenotype (13). M1 macrophages, characterized by enhanced production of pro-inflammatory cytokines, play a dual role in *M. abscessus* infection, while essential for initial pathogen containment, their excessive activation contributes to tissue damage (14, 15). Nucleotide-binding and oligomerization domain-like receptors (NLRs) are members of the pattern recognition receptor (PRR) family, which are mainly expressed in the cytoplasm of macrophages and recognize the signs of pathogenic microorganisms invading cells. NLRs can form inflammasomes, such as NLRP1 (NOD-like receptor protein 1), NLRP3, NLRP4, and AIM2 (absent in melanoma 2) (16). Accumulating studies have highlighted that those receptors participate in signaling pathways and induce the production of pro-inflammatory cytokines, which are crucial for host defense against various pathogens (17). Recent studies by Wang et al. and Zhang et al. have demonstrated the NLRP3 inflammasome’s involvement in macrophage activation and polarization in sepsis (18) and inflammatory conditions (19), though its specific role in *M. abscessus* infection remains poorly understood.

Ouabain, a cardiotonic steroid originally isolated from digitalis species (20), has a well-established history in cardiovascular medicine for treating congestive heart failure and arrhythmias (21). Beyond its cardiac effects, emerging evidence identifies ouabain as an endogenous regulator produced by the adrenal glands and hypothalamus (22). Experimental studies demonstrate ouabain’s ability to modulate inflammatory responses through multiple mechanisms, including downregulation of CD14 (23), CD18 expression (24), and inhibition of the TNF-α/NF-κB pathway (25). While these findings establish ouabain as a promising immunomodulator, its potential role in *M. abscessus* infection remains entirely unexplored.

In this study, we investigate the effects of ouabain on the inflammation induced by *M. abscessus*. Our results show that ouabain significantly attenuates lung inflammation and modulates immune cell infiltration, particularly affecting macrophage populations. Mechanistically, we show that ouabain suppresses M1 macrophage polarization and inhibits NLRP3 inflammasome activation, thereby reducing IL-1β production both *in vivo* and *in vitro*. These results not only contribute to a better understanding of the pathogenesis of *M. abscessus*, but also identify ouabain as a potential therapeutic candidate - either as monotherapy or in combination with existing antibiotics - for the treatment of this clinically challenging infection.

## Materials and methods

### Animals

Female C57BL/6 mice (aged 6–8 weeks, weighing 20  ± 2 g) were purchased from Guangdong Medical Laboratory Animal Center (Guangdong Province, China). All mice were maintained under specific pathogen-free conditions with a 12-hour light/dark cycle at 20–22°C. The animals were allowed free access to drinking water and food. The study complied with the guidelines of the Institutional Animal Care and Use Committees (IACUCs) of the Southern University of Science and Technology and was approved by the Animal Subjects Committee of Shenzhen People’s Hospital (IACUC Number: AUP-220704-LN-0417-01).

### Bacterial culture

*M. abscessus* strain ATCC 410212027 was grown at 37°C on Middlebrook 7H10 agar supplemented with 10% OADC (oleic acid albumin dextrose catalase enriched) and 0.05% Tween-80 (Sigma-Aldrich). *M. abscessus* was collected by centrifugation and resuspended in Middlebrook 7H9 broth, supplemented with 0.2% glycerol, 0.05% Tween 80, and 10% ADC. Middlebrook 7H9 broth, Middlebrook 7H10 agar, OADC, and ADC were purchased from BD Pharmingen (San Diego, CA). The number of CFU bacteria was counted on Middlebrook 7H10 agar.

### Experimental groups and treatments

C57BL/6J mice were randomly assigned to the following groups (12 mice per group): 1) the control group (non-*M. abscessus* infected animals and treated with saline, intraperitoneal injection with saline); 2) *M. abscessus* infected group (animals infected with *M. abscessus* and intraperitoneally injected with saline); and 3) the ouabain-treated group (*M. abscessus* infected animals and intraperitoneal injection with ouabain). *M. abscessus-*infected and ouabain-treated groups were infected with 1.5 × 10^7^ CFU/50 μL *M. abscessus* via intratracheal intubation. An equal volume of saline was administered to the mice in the control group. The animals in the ouabain-treated group were intraperitoneally injected with 0.56 mg/Kg ouabain, based on previous *in vivo* work, for three consecutive days before M. abscessus infection (26). The control group and the M. abscessus-infected group were given equal volumes of saline intraperitoneally for three days. Ouabain octahydrate was purchased from Sigma-Aldrich as a >95% (HPLC) powder (Cat. no. 11018-89-6).

### ELISA analysis

Blood plasma collected from the eyes of mice was used to determine IL-1β, TNFα, IL-6 and IL-10 concentrations using a mouse-specific ELISA kit (Nanjing Jiancheng Bioengineering Institute, China) according to the manufacturer’s instructions. Optical density was measured using a microplate spectrophotometer at 450 nm (VersaMax microplate reader, tunable, BN 2529, Molecular Devices).

### Lung histology

The ligated upper lobe of the left lung was separated, immobilized with 4% paraformaldehyde and dehydrated with alcohol. The lung was then embedded in paraffin and cut into 4 μm thick sections. The tissues were treated with a hematoxylin and eosin kit (Beyotime Biotech, Shanghai, China) and stained and viewed with a light microscope.

### Acid-fast bacillus stain kit (Ziehl–Neelsen’s method)

The paraffin sections were transparent and were dewaxed with solvents (such as petrol and turpentine). The above samples were dehydrated with gradient ethanol and stained with an acid-fast Bacillus (AFB) kit according to the manufacturer’s instructions. Finally, the cells were observed under an oil microscope (Carl Zeiss Jena A1; Carl Zeiss Jena Company, Oberkochen, Germany).

### Immunohistochemical analysis

Sections of mouse lung tissue were deparaffinized and washed with double distilled water. A pre-warmed citrate solution (pH = 6.0) was used for 15 minutes in the microwave for antigen retrieval. After cooling for 30 min, the sections were removed, washed twice with double distilled water (5 min/time) and rinsed three times with 1 × PBS (5 min/time). The endogenous peroxidase was blocked with the endogenous peroxidase blocker (3% H solution) and incubated for 30 min at RT. Then the sections were rinsed three times with 1 × PBS (5 min/time) and incubated for 1 hour with 5% normal donkey serum blocking buffer at RT. The serum was then discarded and the sections were incubated with the primary anti-NLRP3 antibody (Abcam, ab189494, 1:200) at 4°C overnight. Sections were then cleared three times with 1 × PBS (5 min/time) and incubated with HRP-labelled goat anti-rabbit IgG at 37°C in the dark for 2 hours. Sections were then cleared three times with 1 × PBS (5 min/time) and mounted with non-fluorescent buffered glycerol. Finally, the sections were viewed under a fluorescence microscope at high magnification.

### Library construction and high-throughput sequencing and bioinformatic analysis of RNA-seq data (GSE301915)

Fresh mouse lung tissue was selected for RNA sequencing, with three replicates. Total RNA was extracted using TRIzol reagent (15596-026, Life Technologies) according to the manufacturer’s instructions and RNA integrity was assessed using an Agilent Bioanalyzer 2100 (Agilent Technologies). The qualified total RNA was then purified using the RNA Clean XP Kit and the RNase-Free DNase Set. Nine libraries were prepared using the VAHTS Total RNA-seq (H/M/R) Library Prep Kit for Illumina (NR603-02; Vazyme). The library was summarized and sequenced as 150 bp paired-end sequencing reads using an Illumina HiSeq machine. After removing low-quality tags and various contaminants from the original sequencing reads, the clean dataset was processed with mirDeep29 (v2.0.0.5), annotated in the miR-Base database 30 (version 20) as a miRNA reference and aligned with the mouse reference genome (mm10) to predict novel miRNAs. Differentially expressed miRNAs between the four analysis time points were identified by log2Fold-Change| (|log2FC|) ≥1 and a false discovery rate (FDR) <0.05. All predicted target genes were annotated in the database. The web tool DAVID (Visualisation and Integrated Discovery) v6.8 was used for the enrichment analysis of the Gene Ontology (GO) and the Kyoto Encyclopaedia of Genes and Genomes (KEGG) with a significance of FDR <0.05.

### Flow cytometric analysis

According to the instructions for use, single cell suspensions from the lung were prepared and stained as follows: 0.1 μL zombie dye was added to each tube and incubated at room temperature for 10–15 minutes protected from light. The Fc receptor was blocked with CD16/CD32 (Mouse Fc Block, Clone 2.4 G2) for 10 minutes. Mice containing PE anti-CD45 (clone GL-1, Biolegend, San Diego, CA), BV421 anti-mouse F4/80 (clone T45-2342, BD Biosciences, Franklin Lakes, San Diego, CA) and PE-Cy7 anti-mouse CD11b (clone M1/70, BD Biosciences)-specific antibodies that stain extracellular surface markers were combined and stained in the dark for 30 minutes. Unstained cells were used to set the flow cytometer settings. Fluorescence minus one control (FMO) was used to determine the gating limits. Flow cytometric data were acquired using a CYTEK Northern Lights-CLC (Becton and Dickinson, Franklin Lakes, NJ) and analyzed using FlowJo 7.6 software and GraphPad Prism 9.0.

### Transmission electron microscopy

Lung tissue was obtained from the terminal sacrifice of mice and cut into 1–2 mm^3^ cubes. The lung tissue samples and infected cells were fixed with a solution of 2% paraformaldehyde (v/v) and 2.5% glutaraldehyde (v/v) in 0.05 M cacodylate-HCl buffer (pH 7.2) for 2–4 hours. After fixation, the samples were processed using conventional methods. The sections were analyzed using a transmission electron microscope (JEOL, Tokyo, Japan) at an accelerating voltage of 80 kV.

### Isolation and culture of bone marrow-derived macrophages

Mouse bone marrow-derived macrophages (BMDMs) were isolated and differentiated as previously described (26). C57BL/6 mice were sacrificed by dislocation of the spine, bone marrow from the femurs was rinsed and cultured in DMEM (10% FBS + 15% L929 cell-conditioned medium). After 7 days of culture, BMDMs were obtained as a homogeneous population of adherent cells. The number of cells was analyzed with a cell counter and the cells were divided into three groups: Negative control group (same dose of PBS), *M. abscessus* infection group (Multiplicity of Infection (MOI) = 5), and the group treated with Ouabain (the cells were pretreated with ouabain 1 hour before *M. abscessus* infection). 100 nmol/mL was chosen because this concentration was effective in a previous *in vitro* study (27).

### Q-PCR

Total RNA was extracted from lung tissue and cell pellets using TRIzol reagent (Invitrogen, Carlsbad, CA) according to the manufacturer’s instructions. One microgram of total RNA was reverse transcribed using the RevertAid First Strand cDNA Synthesis Kit (Thermo Fisher Scientific, Waltham, MA) to generate cDNA. cDNA was then amplified using the SYBR Green Master Mix (TaKaRa, Mountain View, CA) according to the manufacturer’s protocol. Quantitative real-time PCR was performed using the CFX384 Fluorescent Quantitative Real-Time PCR System (Bio-Rad, Hercules, CA). Relative mRNA concentrations were calculated using the 2-ΔΔCt method, and GAPDH was used as an internal control. The primers used for cDNA synthesis and qPCR are listed in **Supplementary Table S1**.

### Statistical analysis

All statistical analyses were performed using GraphPad Prism software (Prism 9.0). Flow cytometry data were analyzed using FlowJo 10 software. Data are expressed as mean ± SEM, and statistical significance was determined using a two-way analysis of variance (ANOVA) followed by Student’s t-test. Statistical significance was set at *P* < 0.05.

## Results

### Ouabain attenuates pulmonary inflammation and tissue damage in *M. abscessus*-infected mice

**Figure 1:**
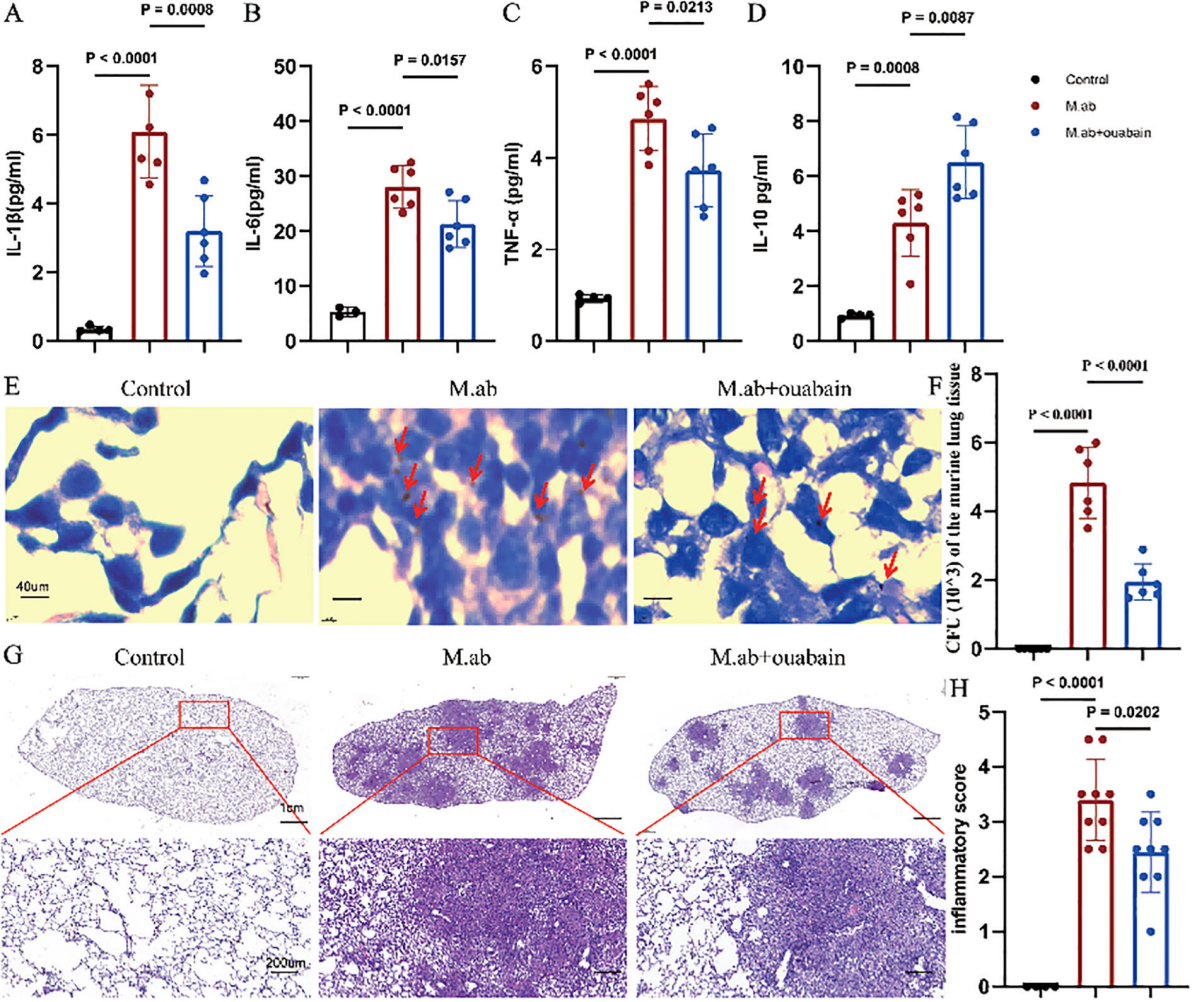
Ouabain attenuates *M. abscessus*-induced pulmonary inflammation. **(A–D)** Plasma cytokine profiles showing ouabain-mediated modulation of inflammatory responses. ELISA quantification of **(A)** IL-1β, **(B)** IL-6, **(C)** TNF-α, and **(D)** IL-10 levels in experimental groups. **(E)** Acid-fast bacilli AFB staining of lung sections (40× magnification; scale bar = 40 μm), with red arrows highlighting mycobacterial clusters. **(F)** Quantitative assessment of pulmonary bacterial burden demonstrating ouabain’s antimicrobial efficacy. **(G)** Representative H&E-stained lung sections at low (2×; scale bar = 1 cm) and high (20×; scale bar = 100 μm) magnification, illustrating pathological improvements with ouabain treatment. **(H)** Histogram quantification of pulmonary inflammation. Data represent mean ± SEM (n = 4-9/group). Control: C57BL/6 mice treated with saline, M.ab: Infected with *M. abscessus* and intraperitoneally injected with saline, *M. abscessus* + Ouabain: Infected with *M. abscessus* and intraperitoneally injected with Ouabain.

To investigate the anti-inflammatory effect of ouabain against M. abscessus infection, we first analyzed the cytokine responses. M. abscessus infection elicited a robust systemic inflammatory response, as evidenced by significantly increased plasma levels of pro-inflammatory cytokines including IL-1β (p < 0.0001, **Figure 1A**), IL-6 (p < 0.0001, **Figure 1B**) and TNF-α (p < 0.0001, **Figure 1C**) compared to saline-treated controls. Interestingly, the anti-inflammatory cytokine IL-10 also showed increased levels post-infection (p < 0.0008, **Figure 1D**). Treatment with ouabain effectively attenuated the cytokine storm triggered by the infection and significantly suppressed the upregulation of pro-inflammatory mediators (p < 0.05, **Figures 1A–C**). Of note, while ouabain reduced overall cytokine production, it maintained elevated IL-10 levels (p < 0.0087, **Figure 1D**), suggesting a possible mechanism for its anti-inflammatory effects. Ouabain treatment significantly reduced the intensity of AFB staining in lung sections (**Figure 1E**) compared to infected controls (no staining was observed in uninfected animals). This correlated with a 2.3-fold decrease in the number of viable bacteria (p < 0.0001, **Figure 1F**), indicating a significant reduction in bacterial burden in the lungs. H&E-stained lung sections showed that M. abscessus infection induced severe granulomatous inflammation with extensive inflammatory infiltration. However, treatment with ouabain reduced the infiltration of inflammatory cells by 58%, maintained alveolar integrity and significantly suppressed the development of granulomas (**Figures 1G, H**). Compared to the saline-treated controls, a separate group of mice received ouabain alone (0.56 mg/Kg), which did not induce systemic toxicity or pulmonary inflammation, consistent with previous reports (28). For the sake of conciseness and to focus on infection-related outcomes, these control data are not presented. Therefore, our results indicate that a successful animal model of *M. abscessus* infection was established, and ouabain exerts potent anti-inflammatory effects in *M. abscessus* pulmonary infection.

### Ouabain reduces pulmonary macrophage and neutrophil infiltration post-infection

**Figure 2:**
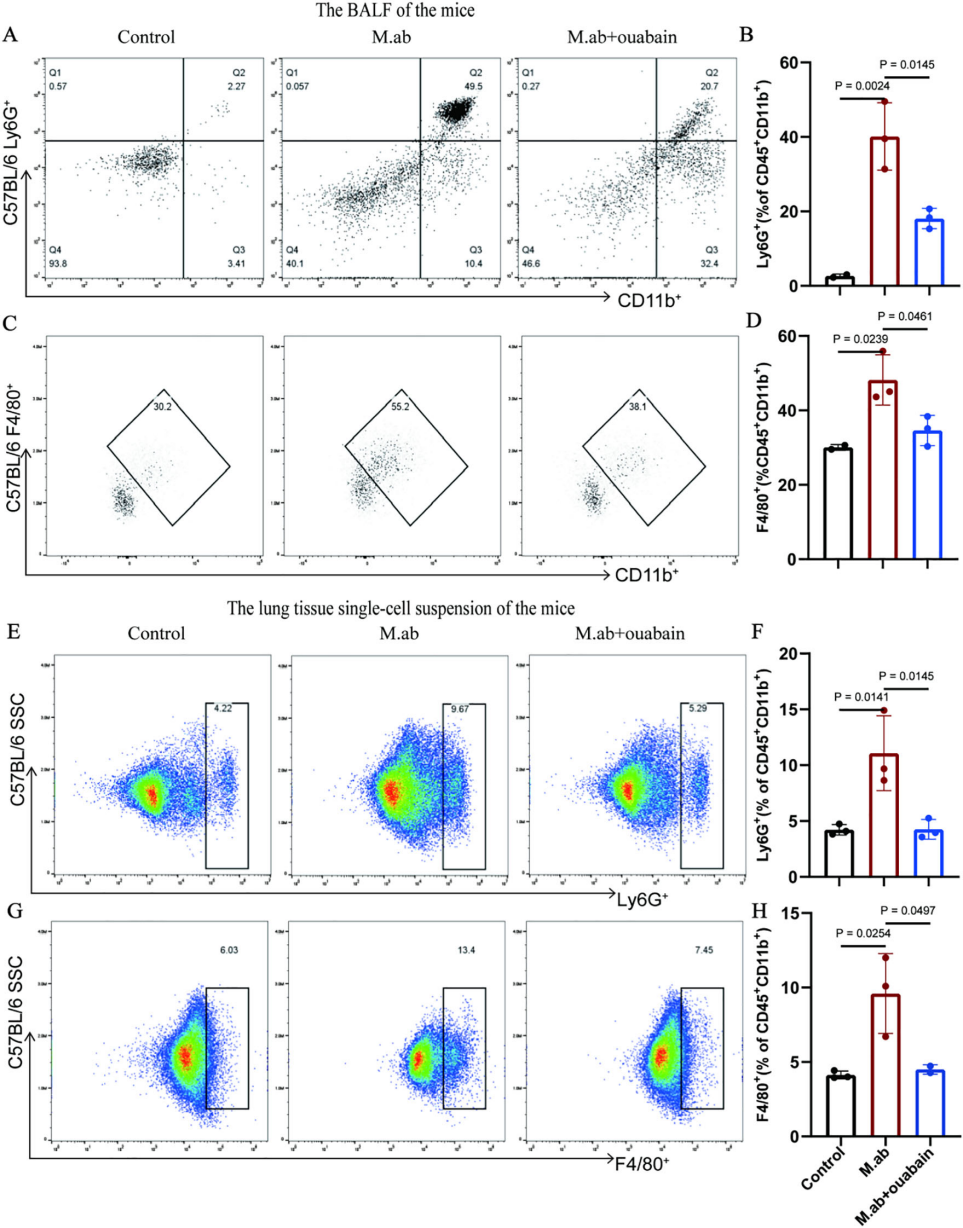
Ouabain modulates neutrophil and macrophage infiltration in *M. abscessus*-infected lungs. **(A)** Representative flow cytometry plots of Ly6G^+^CD11b^+^ neutrophils in BALF. **(B)** Quantitative analysis of neutrophil percentages in BALF. **(C)** Representative FACS plots of F4/80^+^CD11b^+^ macrophages in BALF. **(D)** Quantification of macrophage percentages in BALF. **(E)** Flow cytometry profiles of pulmonary neutrophils. **(F)** Statistical analysis of lung neutrophil infiltration. **(G)** FACS plots of lung macrophages. **(H)** Quantitative assessment of macrophage populations in lung tissue. Data represent pooled results from three independent experiments (mean ± SEM). Groups as in **Figure 1**.

As key mediators of innate immunity, neutrophils and macrophages play crucial roles in host defense against microbial pathogens. To characterize ouabain’s immunomodulatory effects, we performed flow cytometric analysis of leukocyte populations in the *M. abscessus* infection model. We immunostained for double-positive Ly6G/CD11b neutrophils, F4/80/CD11b macrophages, CD11b/CD19 B cells, and CD3/CD8 T cells (**Supplementary Figure S1A**). Gating of BALF CD45^+^ leukocytes, as shown in **Supplementary Figures S1B, C**, and myeloid cells were further analyzed for specific macrophage and neutrophil markers. The percentages of F4/80^+^CD11b^+^ and Ly6G^+^CD11b^+^ subsets showed a significant increase in BALF induced by *M. abscessus* infection compared to the control (*p* < 0.05). However, ouabain treatment decreased the upregulation of the F4/80^+^CD11b^+^ and Ly6G^+^CD11b^+^ subsets in *M. abscessus-infected* lung tissues (*p* < 0.05, **Figures 2A–D**). We also measured the F4/80^+^CD11b^+^, Ly6G^+^CD11b^+^, CD11b^+^CD19^+^, and CD3^+^CD8^+^ subsets in the murine lung tissues. The percentages of neutrophils and macrophages in the ouabain-treated group (5.29% and 7.45%, respectively) were significantly lower than those in the *M. abscessus* infection group (10.72% and 12.5%) (*p* < 0.05, **Figures 2E–H**). We also found that the percentages of CD11b^+^CD19^+^ B cell subsets and CD3^+^CD8^+^ T cell subsets were not significantly different between the *M. abscessus*-infected and ouabain-treated groups (**Supplementary Figures S1D**–**S3G**), which were all lower than those in the control group. These data demonstrate that ouabain specifically modulates innate immune cell recruitment without affecting adaptive immune populations, suggesting targeted regulation of myeloid cell trafficking during *M. abscessus* infection.

### Ouabain suppresses NLRP3 inflammasome pathway activation in *M. abscessus* infection

**Figure 3:**
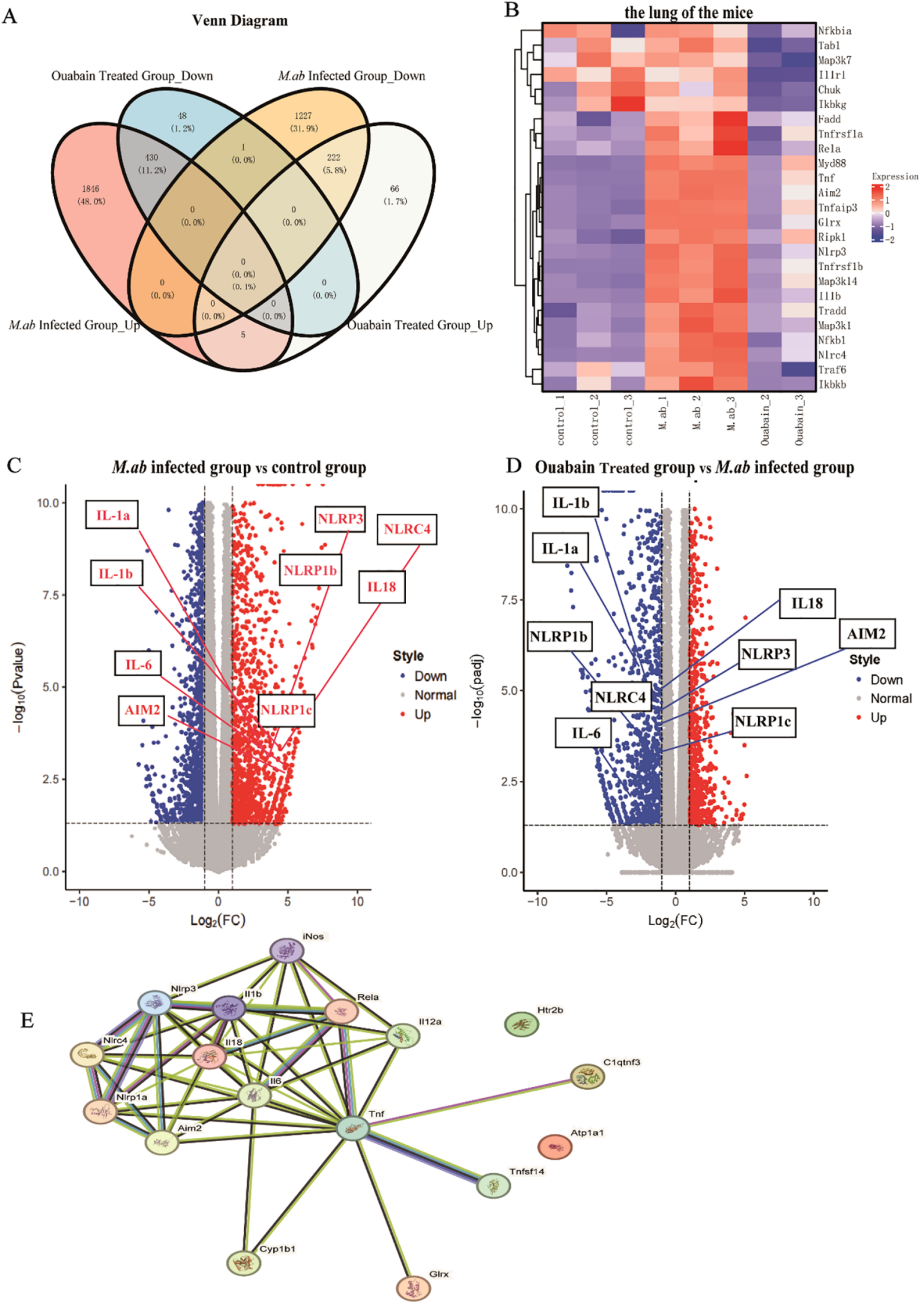
Transcriptomic profiling reveals ouabain-mediated modulation of inflammatory pathways in M. abscessus-infected lungs. **(A)** Venn diagram analysis of differentially expressed genes (DEGs) demonstrating ouabain’s bidirectional regulatory effects. DEGs were identified using thresholds of |log2FC| ≥ 1 and adjusted p-value < 0.05. **(B)** Hierarchically clustered heatmap of significantly regulated inflammatory genes (rows) across experimental groups (columns). The color scale represents z-score normalized expression values, with red indicating upregulation and blue indicating downregulation. **(C, D)** Volcano plots of transcriptomic changes: **(C)** M. abscessus-infected vs control (highlighting infection-upregulated inflammasome genes), **(D)** Ouabain-treated vs infected (showing reversal of inflammatory gene expression). **(E)** Protein-protein interaction network generated using STRING (confidence score > 0.7), highlighting core NLRP3 inflammasome components (red nodes) and their functional associations with key inflammatory mediators (IL-1β, IL-6, TNF-α, iNOS). Edge thickness indicates interaction strength. All data represent biological triplicates (n=3/group). Statistical thresholds: |log2FC| ≥ 1, adjusted p-value < 0.05 (Benjamini-Hochberg correction). Groups as in **Figure 1**.

We performed mRNA transcriptome sequencing to elucidate and identify the possible anti-inflammatory mechanism of ouabain in an *M. abscessus*-infected mouse model. Transcriptome data revealed 3731 DEGs between the control and *M. abscessus*-infected groups, including 1,450 downregulated and 2,281 upregulated genes. 479 downregulated and 293 upregulated genes were identified in the ouabain-treated group (**Supplementary Figure S2A**). Moreover, we observed the intersections of these four gene sets, which generated a Venn plot (**Figure 3A**), indicating that ouabain has a strong and specific regulatory effect on *M. abscessus*-induced inflammation. Remarkably, 89.8% (430/479) of the downregulated genes in the ouabain-treated group were upregulated following *M. abscessus* infection. Conversely, 75.8% (222/293) of the up-regulated genes in the ouabain-treated group were among the *M. abscessus*-infected down-regulated gene sets. The heat map showed that most differentially expressed genes related to the inflammatory response were downregulated in the ouabain-treated group compared with those in the *M. abscessus*-infected group (**Figure 3B**). These genes were highlighted in volcano plots: inflammasome pathway correlation genes were upregulated in the lungs of *M. abscessus*-infected mice compared to the control group (**Figure 3C**). All increases were downregulated after the Ouabain administration (**Figure 3D**). Protein-protein interaction (PPI) analysis was performed using the STRING database (I suppose this is a subheading), which also revealed a strong correlation between the NLRP3 inflammasome and pro-inflammatory factors such as iNOS, IL-1β, IL-6, and TNFα (**Figure 3E**). These results provide compelling evidence that ouabain’s therapeutic effects involve transcriptional regulation of the NLRP3 inflammasome pathway and associated inflammatory mediators during *M. abscessus* infection.

### Ouabain attenuates inflammation through modulation of the NOD-like receptor signaling pathway

**Figure 4:**
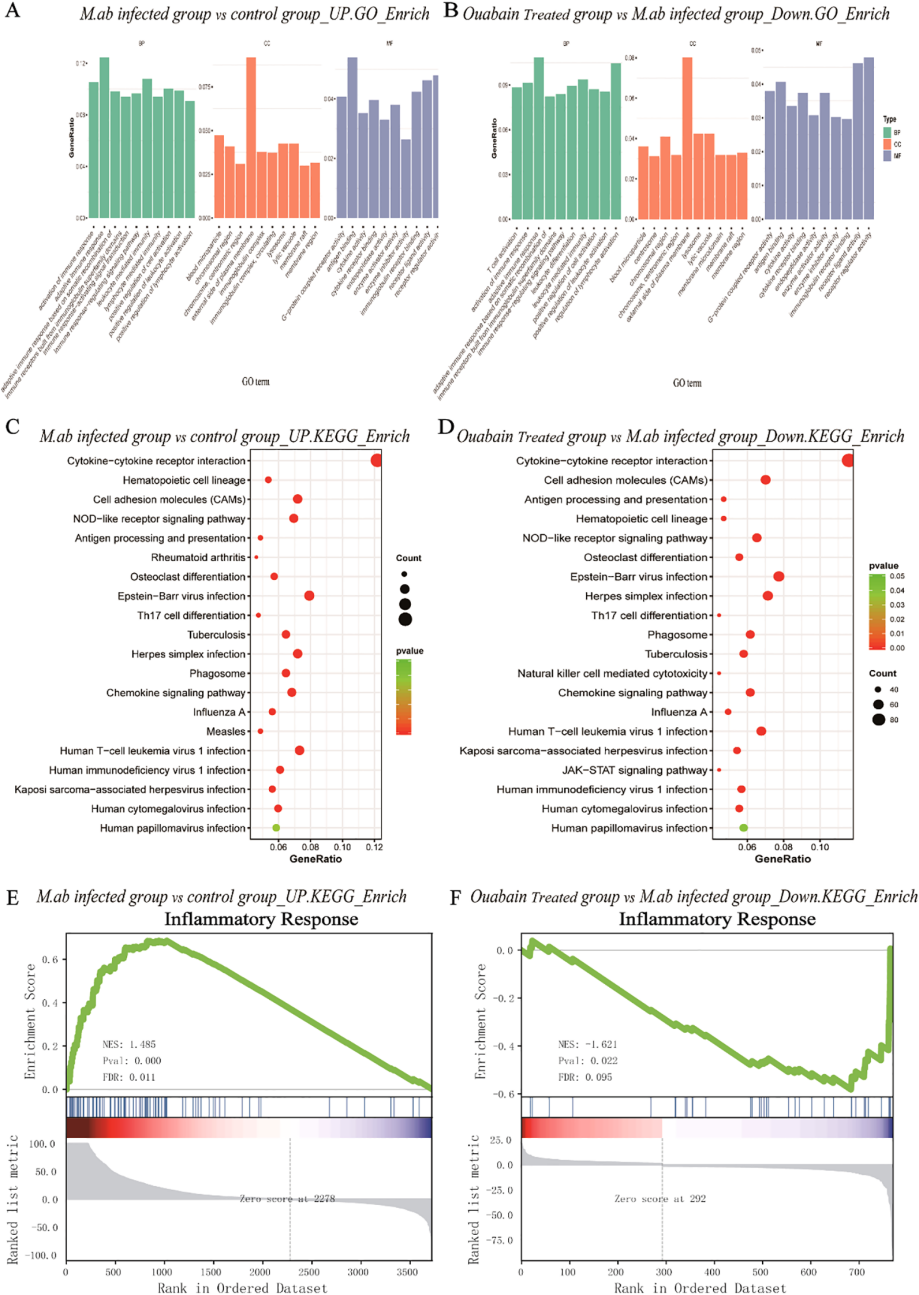
Bioinformatic analysis of transcriptomic alterations in *M. abscessus*-infected lungs following ouabain treatment. **(A)** Upregulated GO terms include biological process, cellular component, and molecular function. **(B)** Down-regulated GO terms include biological process, cellular component, and molecular function. **(C)** KEGG pathway analysis of upregulated proteins. **(D)** KEGG pathway analysis of down-regulated proteins. The vertical axis represents the pathway category, and the horizontal axis represents the enrichment score [−log(P-value)] of the pathway. Significantly enriched KEGG pathways (*P* < 0.05) are presented. GSEA analysis demonstrates that known inflammatory responses are enriched in the M. abscessus-infected model group **(E)**. There is negative regulation of the inflammasome-mediated signaling pathway in the ouabain-treated group **(F)**. Groups as in **Figure 1**.

To determine whether ouabain alleviated the NOD-like signaling pathway induced by *M. abscessus* infection, GO analysis and KEGG pathway enrichment of 222 candidate targets were performed. GO analysis highlighted GO terms with high enrichment of DEGs (>100 genes), which showed that upregulated genes induced by *M. abscessus* were mainly associated with the following functions: response to adaptive immune response (ontology: biological process), external side of the plasma membrane (ontology: cellular component), and receptor regulatory activity (ontology: molecular function) (**Figure 4A**). Depending on the outcome of the *M. abscessus-infected* group, the enriched biological process ontologies in the ouabain-treated group were negative regulation of lymphocyte activation, T cell activation, and activation of the immune response. These enriched molecular functional entities are mainly receptor regulation and receptor-ligand activity. The cell component analysis revealed that the external side of the plasma membrane accounted for the largest proportion (**Figure 4B**). KEGG pathway analysis identified 20 remarkable pathways in the *M. abscessus*-infected group, presenting the pathways of upregulated genes: viral, tuberculosis, phagosome, NOD-like receptor signaling pathway (cellular processes), and immune system (organismal systems) (**Figure 4C**). The enriched KEGG pathways of the ouabain-downregulated genes included the NOD-like receptor signaling pathway, Tuberculosis, Toll-like receptor signaling pathway, NF-κB signaling, and cell adhesion molecules (**Figure 4D**). Given the above results, we submitted the complete gene expression datasets to Gene Set Enrichment Analysis (GSEA) and extracted biological knowledge. Judging from the gene rank distribution and enrichment scores of the rank association matrix in the gene list, both the activated and repressed gene sets showed relatively reliable enrichment. These gene sets were significantly enriched and mainly related to the inflammatory response (**Figures 4E, F**) and cellular response to molecules of bacterial origin (**Supplementary Figures S2B, C**). These findings implied that ouabain has potent, targeted regulatory effects in the *M. abscessus*-infected mouse model, principally influencing infection- and inflammation-associated pathways, such as the NOD-like receptor signaling pathway.

### Ouabain inhibits *M. abscessus*-induced M1 macrophage polarization

**Figure 5:**
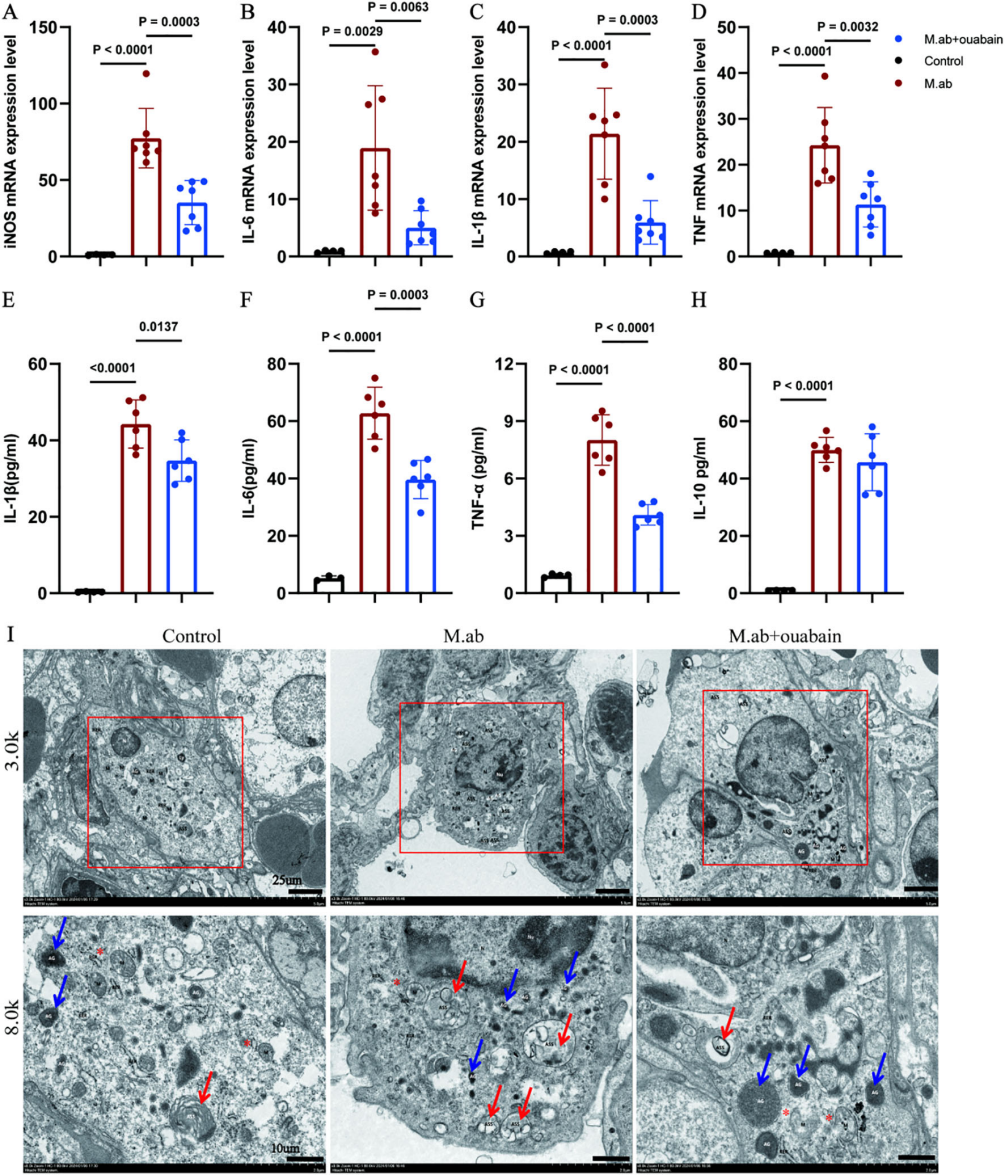
Ouabain suppresses *M. abscessus*-induced M1 macrophage polarization and proinflammatory responses. **(A–D)** qRT-PCR analysis of M1 macrophage markers in lung tissue: Quantification of **(A)** iNOS, **(B)** IL-6, **(C)** IL-1β, and **(D)** TNF-α mRNA expression in *M. abscessus*-infected murine lungs. Data normalized to GAPDH and presented as fold-change relative to uninfected controls (ΔΔCT method). **(E–H)** Circulating cytokine profiles: ELISA measurement of **(D)** IL-1β, **(E)** IL-6, **(F)** TNF-α, and **(G)** IL-10 levels in lung tissue supernatants. Bars represent mean ± SEM. **(I)** Representative images of transmission electron microscopy images in the murine lung tissues in low magnification images (3,000x, Scale bar = 25 μm) and high magnification images (8,000x, Scale bar = 10 μm). All qPCR data represent ≥3 independent experiments performed in duplicate. Groups as in **Figure 1**.

To characterize the immunomodulatory potential of ouabain in *M. abscessus* infection, we first evaluated macrophage polarization patterns in infected murine lungs. Our qPCR results revealed a distinct M1 macrophage polarization profile in response to *M. abscessus* infection. Compared to uninfected controls, infected lung tissues exhibited significant upregulation of key pro-inflammatory markers: inducible nitric oxide synthase (iNOS, 3.8-fold), interleukin-6 (IL-6, 4.2-fold), interleukin-1β (IL-1β, 4.3-fold), and tumor necrosis factor-α (TNF-α, 3.5-fold) (**Figures 5A–D**). Remarkably, ouabain treatment substantially mitigated this inflammatory response, decreasing expression of these markers by more than 2-fold compared to untreated infected mice. These transcriptional changes were corroborated at the protein level. Lung tissues were homogenized in PBS and centrifuged at 12,000 × g for 15 min at 4°C. The resulting supernatants (lung tissue homogenate supernatants) were collected for cytokine analysis. ELISA measurements demonstrated that *M. abscessus* infection significantly elevated lung tissue supernatant concentrations of IL-1β (385 ± 42 pg/mL), IL-6 (320 ± 38 pg/mL), and TNF-α (245 ± 30 pg/mL) (all *p* < 0.001 versus uninfected controls). Ouabain treatment effectively normalized these cytokine levels to 185 ± 22 pg/mL (IL-1β), 160 ± 18 pg/mL (IL-6), and 112 ± 15 pg/mL (TNF-α) (*p* < 0.01 versus infected untreated group; **Figures 5E–G**). Notably, neither infection nor ouabain treatment significantly altered IL-10 levels (**Figure 5H**). Transmission electron microscopy (TEM) analysis demonstrated that *M. abscessus* infection triggered significant activation of pulmonary macrophages, manifested by distinct ultrastructural alterations including: (1) markedly increased cytoplasmic electron density, (2) prominent proliferation of intracellular organelles (particularly mitochondria [M], indicated by red asterisks), and (3) substantial accumulation of both autolysosomes (ASS, red arrows) and azurophilic granules (AG, blue arrows) (**Figure 5I**). Notably, ouabain treatment effectively preserved macrophage ultrastructural integrity, significantly attenuating both organelle stress and the formation of inflammatory particles.

### Ouabain attenuates inflammatory responses by inhibiting NLRP3 inflammasome activation and IL-1β production in macrophages

**Figure 6:**
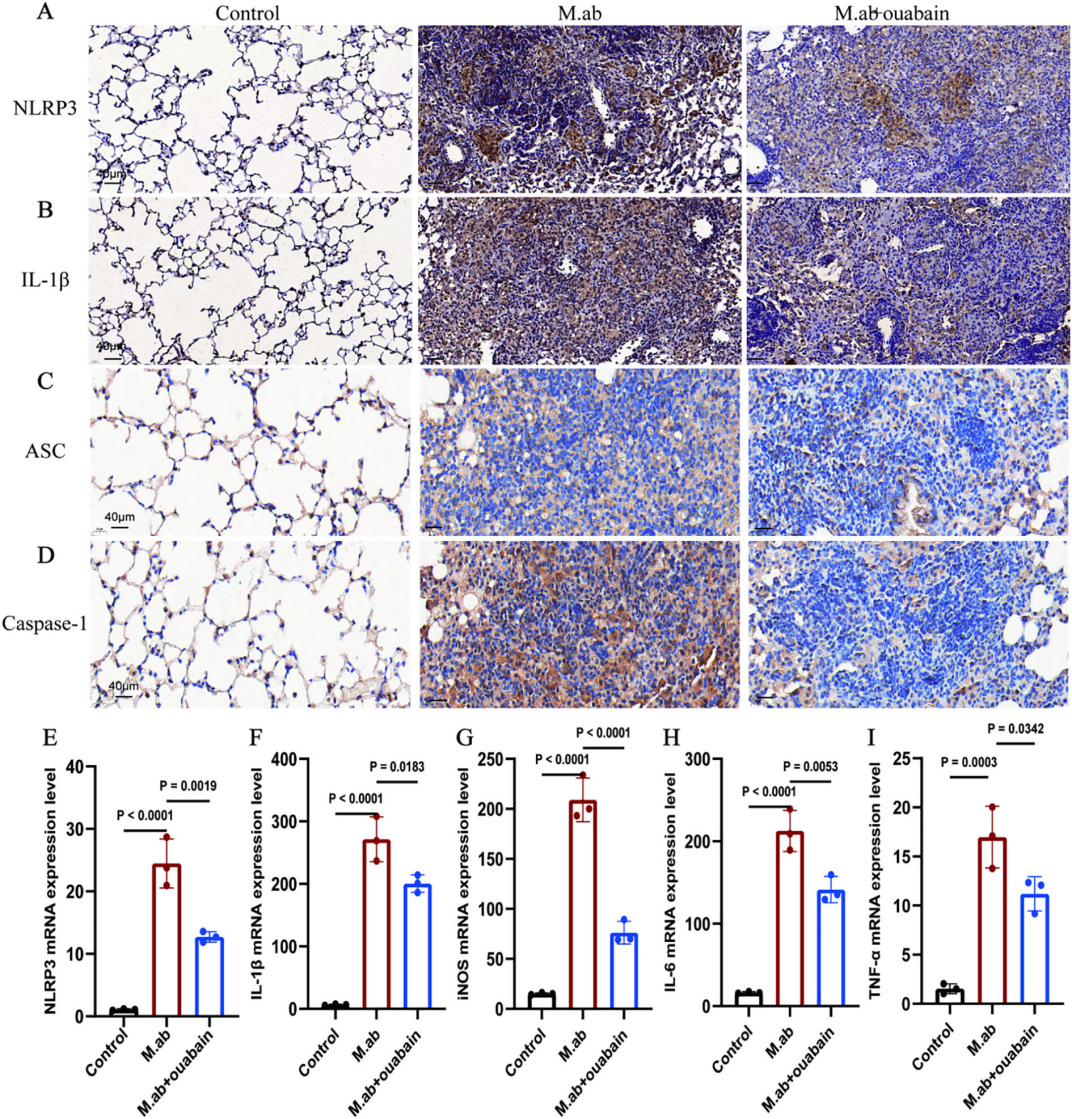
Ouabain inhibits NLRP3 inflammasome activation and IL-1β production in macrophages. **(A)** Representative images of IHC staining for NLRP3 in lung tissue of mice. **(B)** Representative images of IHC staining for IL-1β in lung tissue of mice. **(C)** Representative images of IHC staining for NLRP3 in lung tissue of mice. **(D)** Representative images of IHC staining for IL-1β in lung tissue of mice. Total original magnifications 3×, Scale bar = 500 μm and 20×, Scale bar = 50 μm. **(C–G)** *In vitro* validation using BMDMs: qRT-PCR analysis of **(E)** NLRP3, **(F)** IL-1β, **(G)** iNOS, **(H)** IL-6, and **(I)** TNF-α expression in PMA-differentiated macrophages following *M. abscessus* stimulation and co-treatment with ouabain (10μM). ΔΔCT values were used, and the mRNA expression was corrected for GAPDH and compared to unstimulated murine lung tissues or BMDMs. The qPCR results represent at least three experiments, each set up in duplicate. Groups as in **Figure 1**.

Our transcriptomic analysis identified NLRP3 inflammasome pathway modulation as the principal mechanism underlying ouabain’s anti-inflammatory activity during *M. abscessus* infection. This finding was substantiated by immunohistochemical analysis of lung tissues, which revealed significant downregulation of NLRP3, IL-1β, ASC, and Caspase-1 expression in ouabain-treated animals relative to infected controls (**Figures 6A–D**). These data collectively confirmed ouabain’s suppression of functional inflammasome activation. While ouabain monotherapy significantly reduced inflammation, the incomplete normalization of NLRP3/IL-1β levels suggests two complementary strategies: Low-dose corticosteroids could augment ouabain’s immunomodulation while minimizing toxicity, as shown in tuberculosis models. Following established protocols (27), bone marrow-derived macrophages (BMDMs) were differentiated into M0 macrophages (PMA-stimulated) and challenged with *M. abscessus*. The infection induced pronounced M1 polarization, as demonstrated by 3-4-fold upregulation of characteristic M1 markers (iNOS, IL-6, and TNF-α; *p* < 0.01 versus unstimulated M0 controls, **Figures 6E–I**). Notably, co-treatment with 100 nM ouabain attenuated these pro-inflammatory responses by ≥50% (*p* < 0.05). These findings collectively demonstrate that ouabain mitigates *M. abscessus*-triggered inflammatory responses by specifically inhibiting NLRP3 inflammasome activation and subsequent IL-1β production in macrophages.

## Discussion

Among nontuberculous mycobacteria, *M. abscessus* has gained prominence as a clinically significant pathogen. It causes progressive pulmonary disease that worsens outcomes in patients with underlying lung conditions (29, 30). Our study provides novel insights into the pathogenesis of *M. abscessus* infection and identifies ouabain as a promising immunomodulatory agent against this challenging pathogen.

The pathological features of *M. abscessus* infection share similarities with other mycobacterial infections, including excessive inflammatory cell recruitment, granuloma formation, and subsequent tissue destruction (31). These processes lead to tissue remodeling that compromises lung function and establishes substantial immunological barriers to antimicrobial efficacy (32). Our findings demonstrate that ouabain, a cardiac glycoside traditionally used for heart failure management, exhibits remarkable immunomodulatory properties against *M. abscessus*-induced pulmonary infection. Importantly, our study uncovers novel mechanisms by which ouabain alleviates pulmonary inflammation and diminishes bacterial load, offering promising therapeutic avenues for this notoriously refractory infection. First, we established a robust C57BL/6 mouse model of *M. abscessus* pulmonary infection through intratracheal inoculation with 1.5 x 10^7^ CFU/50 μL, as previously described (33). This well-characterized model recapitulates key features of human disease pathogenesis while providing a standardized platform for evaluating therapeutic interventions. Notably, our study provides the first experimental evidence that ouabain treatment effectively attenuates *M. abscessus*-induced pulmonary inflammation by modulating macrophage polarization dynamics. Specifically, ouabain significantly suppressed M1 macrophage activation, as demonstrated by: (i) marked downregulation of pro-inflammatory cytokines (IL-6, TNF-α, and IL-1β) at both transcriptional and protein levels, (ii) reduced expression of M1 surface markers, and (iii) ultrastructural preservation of macrophage morphology (**Figure 5**). These findings carry important pathophysiological implications, as persistent M1 polarization is known to drive granuloma-associated tissue damage in chronic *M. abscessus* infection (8), impair bacterial clearance through excessive inflammation (6), and contribute to the fibrotic progression observed in clinical cases (9, 34). Mechanistically, we identified NLRP3 inflammasome inhibition as a central component of ouabain’s anti-inflammatory activity. This finding presents an intriguing tissue-specific paradox, as ouabain has been reported to activate NLRP3 in cardiac tissue (35), while our data demonstrate its suppressive effects in pulmonary macrophages. This dichotomy may arise from differential regulation of signaling pathways: suppression of PI3K/Akt-mediated inflammasome priming in macrophages (36) and modulation of Src/MAPK cascades that exhibit tissue-specific expression patterns, cell-type dependent responses. These findings underscore the importance of tissue context when evaluating cardiac glycosides’ immunomodulatory effects, warranting further investigation into cell-specific signaling nodes, dose-response relationships, and potential crosstalk with other inflammasomes. Ouabain demonstrates dual therapeutic efficacy by simultaneously reducing inflammatory pathology and decreasing bacterial burden (**Figures 1F, G**), representing a significant advantage over conventional antibiotics. This combined activity addresses two critical challenges in *M. abscessus* infection management: the limited penetration of antimicrobial agents through granulomatous lesions and bacterial biofilms (37) and the paradoxical worsening of inflammation during antibiotic-mediated bacterial killing. Specifically, our data show that ouabain reduced pulmonary CFU counts by 2.3-fold (*p*<0.0001, **Figure 1F**), attenuated granuloma formation by 58% (**Figures 1G, H**), and preserved alveolar architecture despite infection. This multifactorial action is particularly valuable given *M. abscessus*’s intrinsic resistance to most antibiotics (38), ability to persist within macrophages (12), and rapid development of drug resistance (4, 39, 40). The progression of *M. abscessus* infection is critically driven by chronic inflammation, which not only exacerbates tissue damage but also creates a microenvironment conducive to bacterial persistence. Compounding this challenge, therapeutic options remain severely limited due to the pathogen’s intrinsic antibiotic resistance and remarkable ability to develop adaptive resistance during treatment. Our findings significantly advance the emerging paradigm of host-directed therapy (HDT) by demonstrating that ouabain simultaneously modulates detrimental immune responses through NLRP3 inflammasome inhibition and M1 macrophage polarization, enhances bacterial clearance by maintaining optimal host defense mechanisms (**Figure 1F**), and preserves tissue integrity by preventing inflammation-mediated collateral damage (**Figures 1G, H**). This dual-action mechanism represents a crucial therapeutic balance, sufficiently robust to control infection while avoiding the immunopathology that often undermines conventional antimicrobial strategies. Such immunomodulatory precision aligns with recent HDT approaches for tuberculosis (41) and may be particularly valuable for chronic *M. abscessus infections* in cystic fibrosis patients. Ouabain’s pharmacokinetics and safety profile have been partially characterized in humans, with a plasma half-life of ~18 to 24 hours and dose-dependent effects on cardiac function (20). While its narrow therapeutic window necessitates caution, inhaled or nanoparticle-based delivery systems could mitigate systemic toxicity in pulmonary applications. Recent work with cardiac glycosides in cystic fibrosis models demonstrates that localized delivery (e.g., nebulized ouabain) could enhance lung bioavailability while minimizing systemic exposure (42). The therapeutic use of ouabain is constrained by its cardiotoxicity at high doses, necessitating rigorous dose optimization. Preclinical studies in non-human primates suggest that low-dose ouabain (0.56 mg/Kg) avoids hemodynamic alterations (28), but long-term safety in chronic infections remains unexplored. Although ouabain shows promise as a host-directed therapy, further pharmacokinetic and safety studies—particularly in immunocompromised hosts—are warranted before clinical translation.

Mounting evidence underscores the NLRP3 inflammasome as a critical regulator of antimicrobial host defense, orchestrating immune responses through two key mechanisms: IL-1β maturation, mediated by caspase-1 processing of pro-IL-1β into its bioactive form, and pyroptotic cell death, which triggers Gasdermin-D-dependent release of alarmins and facilitates pathogen clearance (43). This dual functionality enables essential immune defenses, including rapid neutrophil recruitment to infection sites (44), macrophage activation against intracellular pathogens (43), and granuloma formation to contain persistent microbes (45). However, in chronic *M. abscessus* infection, dysregulated NLRP3 activity leads to sustained inflammasome activation, driving tissue damage. Excessive IL-1β promotes fibrotic progression, and impaired resolution exacerbates immunopathology. Our findings reveal that ouabain uniquely modulates this delicate balance by suppressing excessive inflammasome activation (**Figure 6**), maintaining baseline host defense (**Figure 1F**), and preserving tissue architecture (**Figure 1G**). While our data show suppression of NLRP3 expression, ASC oligomerization and caspase-1 cleavage by ouabain, future studies should investigate the direct effects on inflammasome sensor proteins (e.g. NLRP1, NLRC4) and upstream regulators. In our comprehensive *in vitro* screening of macrophage inflammasomes, we systematically evaluated the expression profiles of NLRP3, NLRP1, NLRP6, NLRC4, and AIM2 following *M. abscessus* infection with or without ouabain treatment. Quantitative analysis revealed that NLRP3 exhibited the most significant modulation, with a 4.2-fold induction by infection and 68% suppression with ouabain (*p* < 0.01) (46). Latz et al. reported that NLRP3 is the dominant sensor in mycobacterial infections (43). Ouabain’s known PI3K/Akt modulation pathway (36) that intersects with NLRP3 priming. Our findings demonstrate that ouabain exerts significant immunomodulatory effects on this inflammatory cascade. Specifically, we observed that ouabain treatment suppressed NLRP3 inflammasome activation, reduced secretion of inflammasome-driven IL-1β, and attenuated M1 macrophage polarization both *in vitro* and *in vivo*. These effects contrast with previous reports documenting NLRP3 activation by ouabain in cardiac tissue (35), suggesting tissue-specific modulation of inflammasome activity. Notably, while prior studies established ouabain’s capacity to reduce proinflammatory cytokine production (including IL-1β) in various inflammatory contexts (25, 42, 47), our work provides the first evidence of its specific role in modulating NLRP3 inflammasome activity during *M. abscessus* infection. This novel finding expands our understanding of cardiac glycosides as potential immunomodulators for mycobacterial diseases. The current study makes several important contributions: it reveals tissue-specific effects of ouabain on inflammasome regulation. Provides the first evidence linking ouabain to NLRP3 modulation in mycobacterial infection. However, several mechanistic questions remain unresolved: potential crosstalk between ouabain-sensitive pathways and other inflammasome regulators, tissue-specific determinants of ouabain’s immunomodulatory effects. These knowledge gaps present important opportunities for future research to fully elucidate ouabain’s therapeutic potential against *M. abscessus* and other intracellular pathogens.

## Conclusion

In conclusion, our work establishes ouabain as a multifaceted therapeutic candidate for *M. abscessus* pulmonary disease, with effects spanning both immunomodulation and bacterial control. These findings open new avenues for developing host-directed therapies against challenging NTM infections and provide a strong rationale for further development of ouabain-based therapeutic strategies. While additional preclinical studies are needed, our results offer promising new avenues for addressing the growing clinical challenge of *M. abscessus* infections.

## Data Availability

The datasets presented in this study can be found in online repositories. The names of the repository/repositories and accession number(s) can be found in the article/**Supplementary Material**.
