# Supplementary material for: Ouabain alleviates Mycobacterium abscessus-triggered inflammatory responses through dual regulation of NLRP3 inflammasome activity and M1 macrophage polarization
Source: Front Immunol. 2025 Aug 15;16:1633882. doi: 10.3389/fimmu.2025.1633882 (PMC12394172; doi:10.3389/fimmu.2025.1633882)
Supplement: Supplementary file 1 [file DataSheet1.pdf]

Ouabain alleviates *Mycobacterium abscessus*-triggered inflammatory responses through dual regulation of NLRP3 inflammasome activity and M1 macrophage polarization

Nan Li [a,b,c], Songqiang Huang [a,c], Xing Shi [a], Kuo Lu [a,d], Xiu Yu [a], Chen Qiu [a], Rongchang Chen [a]

Affiliations

[a]The Key Laboratory of Shenzhen Respiratory Diseases, Institute of Shenzhen Respiratory Diseases, The First Affiliated Hospital (Shenzhen People's Hospital), School of Medicine, Southern University of Science and Technology, Shenzhen 518020, China;

[b]College of Food Science and Pharmaceutical Engineering, Zaozhuang University, Zaozhuang 277100, Shandong, China;

[c]Department of Pharmacology, Joint Laboratory of Guangdong–Hong Kong Universities for Vascular Homeostasis and Diseases, School of Medicine, Southern University of Science and Technology, Shenzhen 518055, Guangdong, China;

[d]Henan International Joint Laboratory of Children's Infectious Diseases, Children's Hospital Affiliated to Zhengzhou University, Henan Children's Hospital, Zhengzhou Children's Hospital, Zhengzhou, 450018, China;

* **Corresponding authors at** Institute of Shenzhen Respiratory Diseases, Shenzhen People's Hospital, Shenzhen 518055, Guangdong, China; E-mail addresses: chenrc@vip.163.com (Rongchang Chen)；szchester@163.com(Chen Qiu).

Supplementary results

Table 1: The primer sequences used for the different genes are listed in Table 1 and have been used in our previous studies.

Table 1

Oligonucleotide Primer Sequences for Real-Time Quantitative Polymerase Chain Reaction (qPCR)

gene	sequences (5' to 3')
NOD-like receptor (NLR) family pyrin domain-containing 3 (NLRP3) forward primer	5′-TTCTGCACCCGGACTGTAAA-3′
NOD-like receptor (NLR) family pyrin domain-containing 3 (NLRP3) reverse primer	5′-TCGCCAAGATCATTGTTGCC-3′
NOD-like receptor (NLR) family pyrin domain-containing 1 (NLRP1) forward primer	5′-ACA GAC ATG GAC CTC ATG GTG GTT-3′
NOD-like receptor (NLR) family pyrin domain-containing 1 (NLRP1) reverse primer	5′-CAA CTC CTC CAG GTT TCT GGC TAA-3′
NLR family CARD domain-containing protein 4 (NLRC4) forward primer	5′-ACCTGGAAAAGGATGGGAATGAA-3′
NLR family CARD domain-containing protein 4 (NLRC4) reverse primer	5′-AAGTTTGGCAAGTCCTGGGG-3′
absent in melanoma 2 (AIM2) forward primer	5′-AAA ACT GCT CTG CTG CCT CT-3′
absent in melanoma 2 (AIM2) reverse primer	5′-TCA GCA CCG TGA CAA CAA GT-3′
Interleukin-1beta (IL-1β) forward primer	5′-CCT CAC TGG CAG GAA ATC ATC-3′
Interleukin-1beta (IL-1β) reverse primer	5′-CCT CGT GGA GAC GCT TTA CAT A-3′
Inducible nitric oxide synthase 2 (iNOS) forward primer	5′-CCT GGT ACG GGC ATT GCT-3′
Inducible nitric oxide synthase 2 (iNOS) reverse primer	5′-GCT CAT GCG GCC TCC TTT-3′
Tumor necrosis factor-alpha (TNF-α) forward primer	5′-TCT CAT GCA CCA CCA TCA AGG ACT-3′
Tumor necrosis factor-alpha (TNF-α) reverse primer	5′-ACC ACT CTC CCT TTG CAG AAC TCA-3′
Interleukin-6 (IL-6) forward primer	5′-ATC CAG TTG CCT TCT TGG GAC TGA-3′
Interleukin-6 (IL-6) reverse primer	5′-TAA GCC TCC GAC TTG TGA AGT GGT-3′
Glyceraldehyde 3-phosphate dehydrogenase (GAPDH) forward primer	5′-TGA AGC AGG CAT CTG AGG G-3′
Glyceraldehyde 3-phosphate dehydrogenase (GAPDH) reverse primer	5′-CGA AGG TGG AAG AGT GGG AG-3′

Supplementary Table 2: Complete list of upregulated and downregulated genes between the Mab-infected group and the control group.

Supplementary Table 3: Complete list of up-regulated and down-regulated genes between the ouabain-treated group and the infected group.

Supplementary Figure 1:

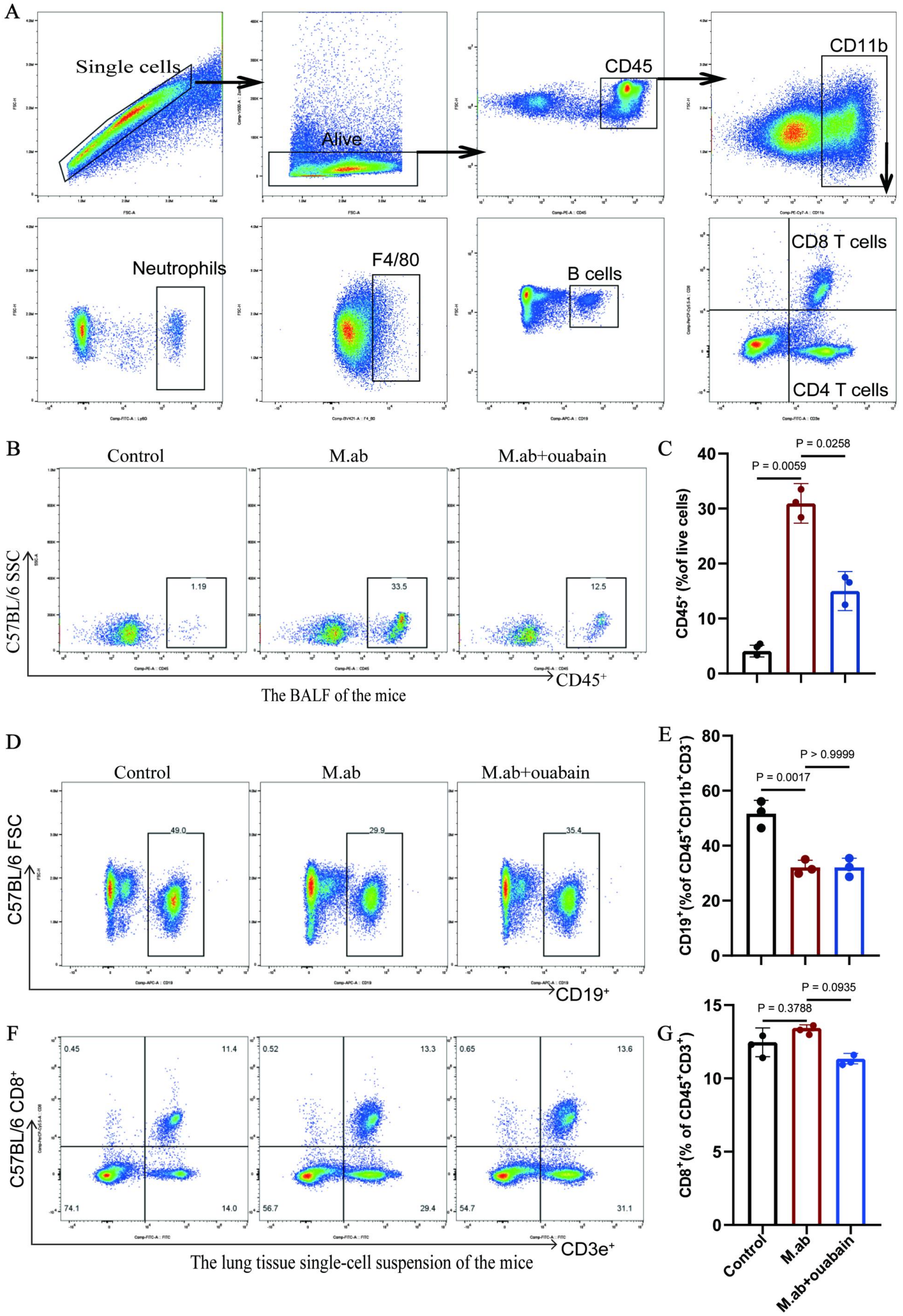

Supplementary Figure 1. Flow cytometry analysis of *M. abscessus* infected murine

model. (A) Gating strategy of flow cytometry analyses to identify nature and acquired immune cells in murine BALF and lung tissues. (B-C) Gating for the BALF CD45^{+} leukocytes. Representative FACS plots (D) and bar graphs (E) show the percentage of B cells in the lung tissues. Representative FACS plots (F) and bar graphs (G) show the percentage of T cells in the lung tissues. Data from three separate experiments were combined and shown. Data are represented as mean ± SEM, * $p < 0.05$ from unpaired student's t-test. ***"Groups as in Fig. 1"*.**

Supplementary Figure 2:

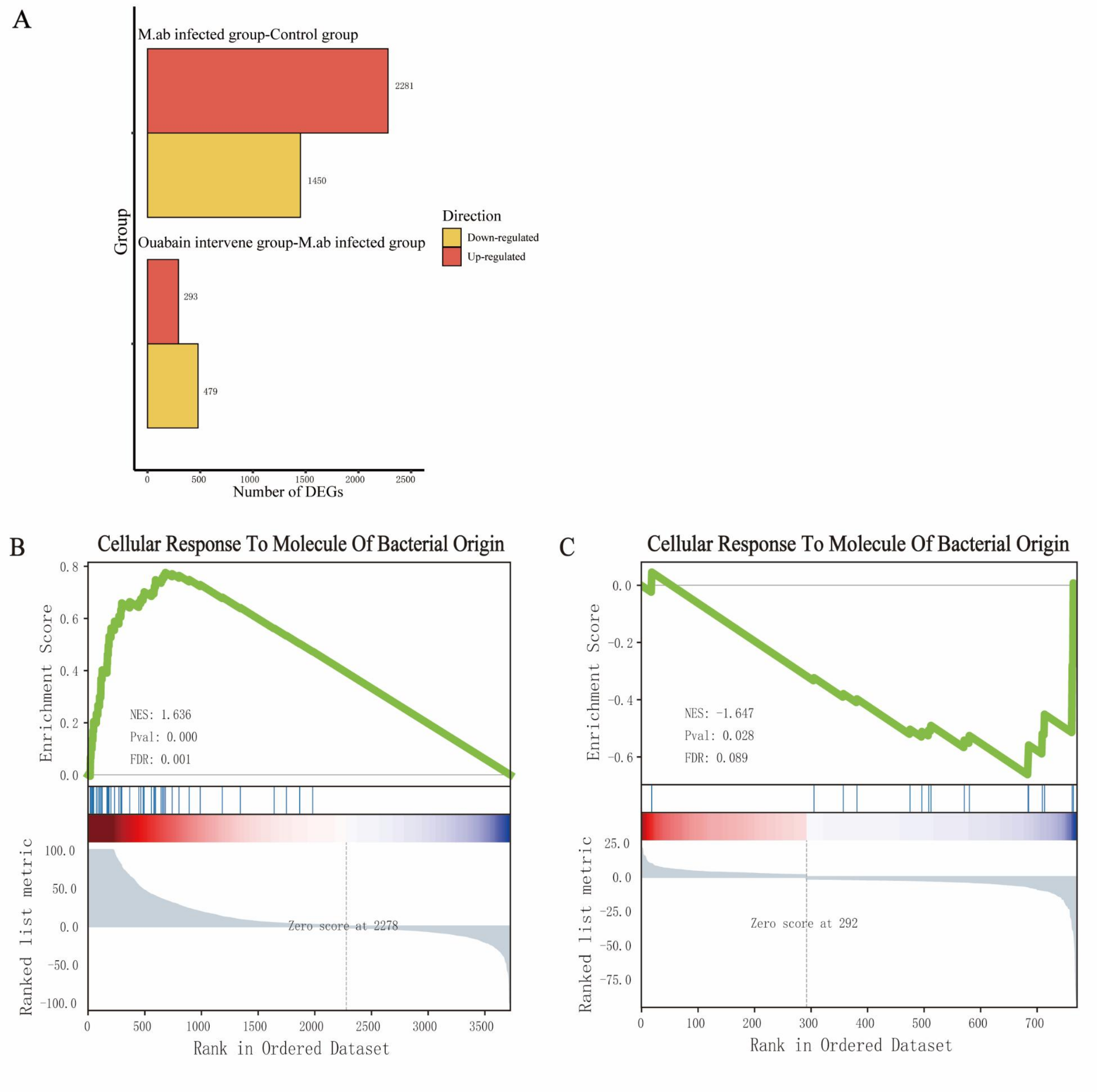

Supplementary Figure 2. RNA-sequencing data analysis of *M. abscessus* infected murine lung tissues (A) The total number of DEGs up-regulated and down-regulated in *M. abscessus* -infected lungs with or without ouabain treatment. GSEA analysis

demonstrates that cellular response to molecules of bacterial origin is enriched in the *M. abscessus* infected model group (B), while negative regulation is in the ouabain treated group (C). *"Groups as in Fig. 1"*.
